# Messung von schmerzbezogener Erlebensvermeidung: Analyse des Acceptance and Action Questionnaire-II-Pain bei Patienten mit chronischem Schmerz

**DOI:** 10.1007/s00482-021-00537-6

**Published:** 2021-02-12

**Authors:** Ronja Majeed, Ira Faust, Michael Hüppe, Christiane Hermann

**Affiliations:** 1 Abteilung Klinische Psychologie und Psychotherapie, Justus-Liebig-Universität Gießen, Otto-Behaghel-Str. 10F, 35394 Gießen, Deutschland; 2 Klinik für Anästhesiologie und Intensivmedizin, Universität zu Lübeck, Ratzeburger Allee 160, 23538 Lübeck, Deutschland

**Keywords:** Schmerzvermeidung, Psychische Flexibilität, ACT, Schmerztherapie, AAQ-II‑P, Pain avoidance, Psychological flexibility, ACT, Pain therapy, AAQ-II‑P

## Abstract

**Einleitung und Fragestellung:**

Erlebensvermeidung („experiential avoidance“) stellt einen zentralen störungsrelevanten Prozess im Rahmen der Akzeptanz- und Commitment-Therapie (ACT) dar. Zur Erfassung wurde der Acceptance and Action Questionnaire II (AAQ-II) entwickelt und in den Niederlanden für eine Patientengruppe mit chronischem Schmerz adaptiert und validiert (AAQ-II‑P). Hohe Werte im AAQ-II‑P bedeuten hohe schmerzbezogene Erlebensvermeidung. Ziel unserer Untersuchung ist die Erfassung von schmerzbezogener Erlebensvermeidung mit einer deutschen Version des AAQ-II‑P bei chronischen Schmerzpatienten und die Prüfung psychometrischer Merkmale des Messverfahrens.

**Methodik:**

Der AAQ-II wurde mittels eines Vorwärts-Rückwärts-Verfahrens ins Deutsche übersetzt, für chronischen Schmerz adaptiert (AAQ-II‑P) und von 168 Patienten einer universitären Schmerzambulanz beantwortet. Zusätzlich wurden Daten zu schmerzbedingter Beeinträchtigung (CPG: Schweregrad nach von Korff) und Schmerzkatastrophisieren (PCS) erhoben sowie zu gesundheitsbezogener Lebensqualität (SF-12), Angst und Depressivität (HADS-D). Ebenfalls erfasst wurden allgemeine Persönlichkeitsmerkmale (BFI‑K) und habituelle Achtsamkeit (KIMS-S). Ausgewertet wurden Reliabilität und faktorielle Validität des AAQ-II‑P sowie seine Beziehung zu den anderen psychometrischen Verfahren.

**Ergebnisse:**

Der AAQ-II‑P erzielte eine hohe interne Konsistenz mit *α* = 0,89 sowie eine eindimensionale Faktorenstruktur mit 61 % aufgeklärter Varianz. Geringe Korrelationen ergaben sich zu Persönlichkeitsdimensionen (maximal *r* = 0,44 zu Neurotizismus) und Achtsamkeit (maximal *r* = −0,43 zu Akzeptanz). Ein hoher Zusammenhang fand sich zu Schmerzkatastrophisieren (*r* = 0,75), Depression (*r* = 0,73) und Angst (*r* = 0,66). Die Beziehung zu Lebensqualität war am stärksten ausgeprägt auf der Psychischen Summenskala (*r* = −0,58).

**Diskussion und Schlussfolgerung:**

Die deutsche Version des AAQ-II‑P hat eine gute Reliabilität und weist hinsichtlich Zuverlässigkeit und Faktorenstruktur hohe Vergleichbarkeit mit der Originalversion auf. Die Beziehungen zu den Skalen der psychometrischen Verfahren sind zumeist in erwarteter Richtung und Höhe. Patienten mit chronischem Schmerz und hoher schmerzbezogener Erlebensvermeidung tendieren deutlich zum Schmerzkatastrophisieren und zeichnen sich durch schlechtere psychische Lebensqualität aus. Dies spricht für die Relevanz des Konstrukts hinsichtlich therapeutischer Zielvariablen.

## Abstract (en)

**Introduction and objective:**

Experiential avoidance is a core process variable in the concept of Acceptance and Commitment Theory (ACT) and has been connected to various disorders. A widely used instrument for the measurement of experiential avoidance is the Acceptance and Action Questionnaire II (AAQ-II). Recently, a pain-specific version for chronic pain patients was introduced in the Netherlands (AAQ-II‑P). High scores indicate strong pain-related experiential avoidance. The aim of the current study was to measure pain-related experiential avoidance in a sample of chronic pain patients using the German translation of the AAQ-II‑P and to analyze its psychometric qualities.

**Methods:**

After a forward-backward translation procedure of the AAQ-II and adaptation to the pain context, a sample of *N* = 168 patients from a multidisciplinary pain center answered the German version of the AAQ-II‑P. Additional questionnaires were administered to measure specific constructs of interest: chronic pain grade (CPG), pain catastrophizing (PCS), health-related quality of life (SF-12), psychopathology (HADS-D), personality (BFI‑K) and mindfulness (KIMS-S). Reliability, factorial validity and construct validity of the AAQ-II‑P were identified.

**Results:**

The AAQ-II‑P had high internal consistency (*α* = 0.89) and the one-factor solution explained 61% of the total variance. Correlations to personality and mindfulness subscales were low (maxima: *r* = 0.44 with neuroticism and *r* = −0.43 with acceptance). High correlations were shown for catastrophizing (*r* = 0.75), depression (*r* = 0.73) and anxiety (*r* = 0.66). Also, there was a substantial correlation to health-related quality of life, specifically the psychological total scale (*r* = −0.58).

**Discussion and conclusion:**

The German AAQ-II‑P has good reliability. Regarding factorial structure and construct validity, it is highly comparable to the original version. Direction and magnitude of the relationship to adjacent constructs mostly measure up to our expectations. Chronic pain patients inclined to pain-related experiential avoidance tend to indicate more pain catastrophizing and show restricted quality of life regarding psychological variables. Apparently, pain-related experiential avoidance can be relevant for popular target variables in psychotherapy.

Für das Verständnis und die Behandlung chronischer Schmerzen haben Konzepte der ACT in den letzten Jahren an Bedeutung gewonnen. Besonders Erlebensvermeidung rückt als störungsrelevanter Prozess in den Fokus. Zur Quantifizierung wird häufig der AAQ-II verwendet, von dem nun auch eine schmerzspezifische Variante vorliegt (AAQ-II‑P). Schmerzbezogene Erlebensvermeidung umfasst Verhaltensweisen, Kognitionen und Emotionen, die darauf abzielen, Schmerzerleben in jeglicher Facette zu vermeiden und zu kontrollieren. In diesem Beitrag wird eine deutsche Version des AAQ-II‑P vorgestellt und die psychometrischen Gütekriterien des Verfahrens werden überprüft.

## Einleitung und Fragestellung

Erlebensvermeidung („experiential avoidance“) und Akzeptanz („acceptance“) sind zentrale Prozesse im Rahmen der Akzeptanz- und Commitment-Therapie (ACT). Unter Erlebensvermeidung werden Anstrengungen und Bemühungen verstanden, die darauf abzielen, negativ bewertete Gefühle, Gedanken, Vorstellungen oder Erinnerungen an eine Situation oder einen Zustand zu vermeiden oder zu kontrollieren [15]. Den Gegenpol bildet Akzeptanz: Auch aversive Gedanken und Gefühle werden bereitwillig von Moment zu Moment akzeptiert und das Verhalten entsprechend persönlichen Werten situationsabhängig beibehalten oder verändert [51]. Beide Prozesse sind eingebettet in das konzeptionell weiter gefasste Konstrukt der psychologischen (In‑)Flexibilität [8], werden jedoch teilweise auch synonym verwendet [3, 48]. Sie werden in den letzten Jahren verstärkt im Kontext von chronischen Schmerzerkrankungen diskutiert [21, 37]. Aktuelle Studien bezeugen die Relevanz als Moderatorvariable bei schmerztherapeutischen Interventionen [11, 45].

Neuere Studien bekunden jedoch zunehmend Zweifel an der Konstruktvalidität des AAQ-II, insbesondere der hohe Zusammenhang zu dispositionellen Belastungsindikatoren wie Neurotizismus wird kritisiert [10, 48, 54, 57]. Unklarheiten in der Definition des untersuchten Konstrukts kommen verschärfend hinzu: So wird ein und dasselbe Instrument je nach Fragestellung zur Messung von Erlebensvermeidung, Akzeptanz oder psychischer (In‑)Flexibilität herangezogen.

Bei der Untersuchung von schmerzbezogenen wissenschaftlichen und therapeutischen Fragestellungen könnte eine Eingrenzung des Konstrukts auf den Schmerzkontext definitorische Klarheit bringen und die Testgüte erhöhen. Andere situations- und symptomspezifische Varianten des AAQ-II sind bereits etabliert.

Reneman et al. definieren schmerzbezogene Erlebensvermeidung („experiential avoidance of pain“) als jegliches offene oder verdeckte Verhalten, das darauf abzielt, Schmerz in jeglicher Facette zu vermeiden oder zu kontrollieren [47]. Das schließt neben dem Schmerzerleben auch schmerzbezogene Gedanken, Emotionen und Kognitionen ein. Erfolgreiche Erlebensvermeidung von Schmerz ist kurzfristig mit positiven Konsequenzen verbunden (z. B. Entlastung), die über negative Verstärkung auf die Beibehaltung von Erlebensvermeidung wirken. Längerfristig sind jedoch negative Folgen wahrscheinlich, da das Verhaltensrepertoire rigide auf Vermeidung ausgerichtet ist und eine situationsadäquate Verhaltensvariabilität fehlt. Schmerzakzeptanz bezeichnet „das Bemühen, das eigene Funktionsniveau trotz bestehender Einschränkungen zu erhalten, sowie die Tendenz, Schmerzen nicht um jeden Preis vermeiden zu wollen“ [41]. Schmerzakzeptanz beinhaltet damit die Fähigkeit, trotz Schmerzen entsprechend persönlichen Werten und den sich daraus ergebenden Zielen flexibel handeln zu können. Ein gebräuchliches Erhebungsinstrument für Schmerzakzeptanz ist der Chronic Pain Acceptance Questionnaire (CPAQ), in dem das Interesse an positiven Alltagsaktivitäten und der Verzicht auf anhaltenden Kampf gegen den Schmerz erhoben werden. Von der revidierten Form [37] liegt eine validierte deutsche Version vor [41].

### Erfassung von Erlebensvermeidung

Zur Erfassung von Erlebensvermeidung entwickelten Hayes et al. den Acceptance and Action Questionnaire (AAQ; [16]), der von Bond et al. wegen inkonsistenter psychometrischer Kennwerte modifiziert wurde und die Bezeichnung AAQ-II erhielt [3]. Der AAQ-II besteht aus 7 Items, die mittels einer 7‑Punkte-Likert-Skala (Itemwerte 1–7) zu beantworten sind. Aus der Summe der Einzelitems ergibt sich ein Gesamtwert. Die Auswertung des AAQ-II ist in der Literatur nicht einheitlich. Mehrfach wird das mit dem AAQ-II erfasste Konstrukt als psychologische Flexibilität („psychological flexibility“) bezeichnet, die in geringer Ausprägung durch Erlebensvermeidung und in hoher Ausprägung durch Akzeptanz gekennzeichnet ist. In Studien mit Betonung der Erlebensvermeidung werden die Itemwerte wie angekreuzt aufsummiert, sodass ein hoher Skalenwert hohe Erlebensvermeidung (hohe psychologische Inflexibilität) anzeigt [3, 31, 47]. Dagegen werden in Studien mit Betonung des Akzeptanzbegriffs die Itemwerte üblicherweise invertiert, sodass eine hohe Skalensumme hohe Akzeptanz (psychologische Flexibilität) charakterisiert [12, 20, 32, 35, 51]. Akzeptanz bedeutet dabei nicht, Belastungen wie z. B. Schmerzen „einfach hinzunehmen“ (zu akzeptieren), sondern trotz und mit diesen Belastungen entsprechend eigenen Werten und Zielen agieren zu können. Damit ist das Konzept über ACT hinausgehend relevant [20, 26, 36].

Untersuchungen zum AAQ-II belegen eine einfaktorielle Struktur, gute Reliabilität (Cronbachs α: 0,78–0,88), Veränderungssensitivität und deutliche theoriekonforme Beziehungen zu Merkmalen psychischer Belastung (Angst, Depression, Global Severity Index: Korrelationen zwischen 0,49 und 0,71; [3]). Die psychometrischen Merkmale und genannten Beziehungen fanden sich auch bei einer deutschen Version des AAQ-II (Fragebogen für Akzeptanz und Handeln: FAH-II [12]) sowie bei einer Stichprobe chronischer Schmerzpatienten [38].

Reneman et al. adaptierten den AAQ-II [3] für Patienten mit chronischen Schmerzen [47]. Inhaltlich wurden dafür die Items schmerzbezogen formuliert (z. B. original: *„I’m afraid of my feelings“*, neu*: „I’m afraid of my pain“*). Der Antwortmodus blieb identisch. Geringe Werte sprechen für hohe Schmerzakzeptanz (schmerzbezogene psychologische Flexibilität) und hohe Werte für ausgeprägte Erlebensvermeidung von Schmerz (schmerzbezogene psychologische Inflexibilität). Das Verfahren wurde AAQ-II‑P benannt und bei Patienten mit chronischen Schmerzen in niederländischen Rehabilitationseinrichtungen eingesetzt. Die Ergebnisse sprechen für ein eindimensionales Messverfahren mit guter Reliabilität (*α* = 0,87), das geringe bis mittelstarke Beziehungen zu Schmerzkatastrophisieren und schmerzbezogener psychologischer Rigidität aufweist. Die Autoren nahmen eine mittelstarke bis starke Beziehung zu gesundheitsbezogenem psychischem und körperlichem Befinden an, fanden hier aber nur schwache Zusammenhänge [47].

### Untersuchungsziel und Hypothesen

Ziel unserer Untersuchung ist die Erfassung von schmerzbezogener Erlebensvermeidung mit einer deutschen Version des AAQ-II‑P bei chronischen Schmerzpatienten und die Prüfung psychometrischer Merkmale des Messverfahrens.

Vergleichbar mit Reneman et al. [47] formulierten wir für die deutsche Fassung des AAQ-II‑P die Hypothesen,

1. dass die Items des AAQ-II‑P ein eindimensionales Merkmal erfassen und es reliabel abbilden,
2. dass der Summenscore des AAQ-II‑P schwach oder mittelstark korreliert mit Skalen anderer psychometrischer Verfahren, die mit dem Konzept der schmerzbezogenen Erlebensvermeidung vergesellschaftet sind (z. B. positive Beziehung zu Schmerzkatastrophisieren; negative Beziehung zu Achtsamkeit, speziell zu Akzeptanz),
3. dass der Summenscore des AAQ-II‑P positive Beziehungen zu Merkmalen aufweist, die Schmerzintensität, schmerzbedingte Beeinträchtigung, Angst, Depressivität und schlechte gesundheitsbezogene Lebensqualität kennzeichnen und die für die Dokumentation chronischer Schmerzpatienten und für die Qualitätssicherung schmerzbezogener Interventionen empfohlen werden [6, 22–24].
4. Ergänzend nahmen wir an, dass der Summenscore des AAQ-II‑P nur schwache Beziehungen zu psychologischen dispositionellen Persönlichkeitsdimensionen aufweist. Diese Hypothese basiert auf der Annahme, dass der AAQ-II‑P ein Einstellungsmuster reflektiert, das grundsätzlich änderungssensitiv ist und sich damit von Persönlichkeitsdimensionen mit hoher Zeit- und Situationskonstanz unterscheidet.

## Methodik

### Rekrutierung der Patientenstichprobe

Nach positiven Voten der Ethikkommissionen der Universität Gießen und der Universität zu Lübeck nahmen an der Untersuchung Patienten teil, die sich zum Befragungszeitpunkt wegen chronischer nichtmaligner Schmerzen in Behandlung der Schmerzambulanz der Klinik für Anästhesiologie und Intensivmedizin des UKSH Campus Lübeck befanden. Einschlusskriterien waren ein Alter ab 18 Jahren und keine in der Patientenakte vermerkte psychiatrische Erkrankung.

Von 258 für die Rekrutierung geeigneten Patienten waren 30 telefonisch nicht erreichbar. 228 Patienten wurden telefonisch kontaktiert und über das Studienvorhaben informiert. 25 Patienten (11 %) lehnten eine Teilnahme ab. 203 an der Studie interessierte Patienten erhielten postalisch schriftliches Aufklärungsmaterial sowie das Fragebogenpaket, das sie in einem vorfrankierten Umschlag zusammen mit einer Einwilligungserklärung zurückschicken konnten. Es nahmen letztlich 171 Patienten an der Studie teil. Von 168 Patienten lagen Daten zum AAQ-II‑P vor. Die Patienten dieser Analysestichprobe waren im Durchschnitt 52,3 Jahre alt (SD = 10,2; Range: 19–70 Jahre), mehrheitlich weiblich (77,4 %) und hatten mehrheitlich einen Haupt- oder Realschulabschluss (76,2 %).

### Fragebögen

Folgende Fragebögen kamen zur Anwendung.

#### Deutsche Version des AAQ-II‑P

Der Acceptance and Action Questionnaire-II wurde über die Prozedur der Übersetzung-Rückübersetzung mithilfe von zwei Muttersprachlern in eine deutsche Version gebracht. Anschließend wurde analog zum Vorgehen der niederländischen Forschergruppe der Itemwortlaut an den Schmerzkontext angepasst (z. B. Item 2: „Ich habe Angst vor meinen Schmerzen“; Item 7: „Sorgen über Schmerzen stehen meinem Erfolg im Weg“). Das Verfahren hat sieben Items, die mit einer 7‑Punkte-Likert-Skala (Werte 1–7) zu beantworten sind. Ausgewertet wird der Summenwert der Items. Die Auswertung erfolgte analog zu Reneman et al. [47], sodass hohe AAQ-II‑P-Summenwerte hohe schmerzbezogene Erlebensvermeidung und schmerzbezogene Inflexibilität ausdrücken. Geringe Werte kennzeichnen demnach eine hohe schmerzbezogene Akzeptanz.

#### Chronic Pain Grade Questionnaire (CPG)

Der Chronic Pain Grade Questionnaire [29] dient der Graduierung chronischer Schmerzen. Eingesetzt wurde die validierte deutsche Version des Verfahrens [27]. Zur Berechnung des Schweregrads werden Angaben zur Schmerzintensität (aktuelle, durchschnittliche, größte Schmerzstärke) und Angaben zur schmerzbedingten Beeinträchtigung der letzten drei Monate verwendet (Alltag, Freizeitaktivitäten, Arbeitsfähigkeit). Diese werden mittels numerischer Rating-Skalen (0–10) eingeschätzt. Zusätzlich erfolgt eine Angabe, an wie vielen Tagen in den letzten drei Monaten aufgrund von Schmerzen nicht der üblichen Aktivität nachgegangen werden konnte. Aus den Angaben werden vier Schmerzgrade nach von Korff berechnet: geringe Beeinträchtigung und geringe Schmerzstärke (1), geringe Beeinträchtigung und hohe Schmerzstärke (2), mittelstarke Beeinträchtigung – unabhängig von Schmerzstärke (3), starke Beeinträchtigung – unabhängig von Schmerzstärke (4). Zusätzlich zu der kategorialen Auswertung wurde die typische Schmerzintensität als Mittelwert der Angaben zur aktuellen, durchschnittlichen und größten Schmerzstärke berechnet sowie die typische schmerzbedingte Beeinträchtigung als Mittelwert der Skalen zur Beeinträchtigung im Alltag, in der Freizeit und bei der Arbeitsfähigkeit.

#### Kentucky Inventory of Mindfulness Skills – Short version (KIMS-S)

Das KIMS‑S ist eine Kurzform des Kentucky Inventory of Mindfulness Skills [1]. Es ist ein Verfahren zur multidimensionalen Quantifizierung von Achtsamkeit. Die Kurzform KIMS‑S enthält 20 Items, die mittels einer 5‑Punkte-Likert-Skala zu beantworten sind (Werte 1–5). Erfasst werden die Subskalen „Beobachten“, „Beschreiben“, „Mit Aufmerksamkeit handeln“ und „Akzeptieren ohne Bewertung“ [19].

#### Pain Catastrophizing Scale (PCS)

Die PCS [53] erfasst mit 5‑fach abgestuften Items (Werte 0–4) 13 Gedanken und Gefühle, die man im Zusammenhang mit Schmerzen hat. Ausgewertet werden die Subtests „Rumination“, „Magnifikation“ und „Hilflosigkeit“ sowie ein Gesamtwert. Angewandt wurde die validierte deutsche Version [39].

#### Hospital Anxiety and Depression Scale (HADS-D)

Die HADS‑D [18] erfragt mittels 14 Items über 4‑stufige Likert-Skalen (Werte 0–3) das Befinden der letzten Woche. Sieben Items betreffen das Merkmal „Depression“ und sieben Items betreffen das Merkmal „Angst“. Gebildet werden Summenwerte, acht Items müssen zuvor umcodiert werden.

#### Gesundheitsbezogene Lebensqualität (Short Form-12)

SF-12 ist die Kurzform von SF-36 und ein krankheitsübergreifendes Messinstrument zur Erfassung der gesundheitsbezogenen Lebensqualität bezogen auf die letzten vier Wochen. Acht Dimensionen der subjektiven Gesundheit (körperliche Funktionsfähigkeit, körperliche Rollenfunktion, körperliche Schmerzen, allgemeine Gesundheitswahrnehmung, Vitalität, soziale Funktionsfähigkeit, emotionale Rollenfunktion und psychisches Wohlbefinden) lassen sich den zwei Grunddimensionen „körperliche Gesundheit“ und „psychische Gesundheit“ zuordnen [40].

#### Big Five Inventory – Kurzform (BFI‑K)

Auf Grundlage des prominenten Fünf-Faktoren-Modells der Persönlichkeit wurden die Dimensionen „Extraversion“, „Verträglichkeit“, „Gewissenhaftigkeit“, „Neurotizismus“ und „Offenheit für Erfahrungen“ mittels einer Kurzform des Big Five Inventory erfasst [46]. Das Verfahren hat 21 Items, die mittels 5‑stufiger Likert-Skala zu beantworten sind (Werte 1–5), die Skalenbildung erfolgt über den Mittelwert.

### Klinische Merkmale

Als klinische Merkmale wurden der Patientenakte die Hauptschmerzlokalisation und das Schmerzchronifizierungsstadium nach dem Mainzer Stadienmodell [14] entnommen. Beide Merkmale wurden mit dem Schmerzdokumentationssystem QUAST erfasst [13]. Die vom Patienten beklagte Schmerzsymptomatik wird dabei entsprechend der IASP-Taxonomie mithilfe von MASK‑S einer von insgesamt 6 Hauptdiagnosegruppen zugeordnet [28]. Die Einstufung der Chronifizierung nach dem Stadienmodell basierte auf ärztlicher Anamnese.

### Auswertung

Zur Prüfung der Dimensionalität des AAQ-II‑P wurde eine Faktorenanalyse (Hauptkomponentenanalyse mit Varimax-Rotation) berechnet. Außerdem wurde eine Itemanalyse durchgeführt und die Reliabilität ermittelt (interne Konsistenz nach Cronbachs α). Zur Überprüfung der Normalverteilung des AAQ-II‑P-Summenwerts wurde der Kolmogorow-Smirnow-Test gerechnet.

Die Konstruktvalidität wurde durch Pearson-Produkt-Moment-Korrelationen zu den Skalen der anderen psychometrischen Verfahren bestimmt. Eine Prüfung auf Normalverteilung erfolgte mit Ausnahme des AAQ-II‑P nicht, da die übrigen psychometrischen Verfahren nicht Gegenstand der Testgütebestimmung waren, die Normalverteilung keine notwendige Bedingung für die Korrelationsberechnung ist und deren Signifikanzbestimmung bei hinreichend großem Stichprobenumfang robust gegen Verletzung der Normalverteilung ist und dabei konservativ ausfällt [4].

Zur besseren Vergleichbarkeit der Ergebnisse wurde die Bewertung der Korrelationen von Reneman et al. [47] übernommen: ab 0,75 starker Zusammenhang; 0,50 bis < 0,75 mittlerer Zusammenhang; 0,25 bis < 0,50 geringer Zusammenhang; < 0,25 kein Zusammenhang. Die semantische Interpretation der Korrelationshöhe im Sinne einer Effektstärke ist in der Literatur uneinheitlich und muss unter Berücksichtigung der verglichenen Merkmale und ihrer Erfassungsmethoden gesehen werden [50]. Für psychometrische Verfahren erscheinen die Empfehlungen von Cohen [7] zur Charakterisierung der Höhe von Korrelationen vor dem Hintergrund der durch sie ausgedrückten gemeinsamen Varianz nur bedingt geeignet (mittelstarker Zusammenhang ab 9 % gemeinsamer Varianz, starker Zusammenhang ab 25 % gemeinsamer Varianz). Die von Reneman et al. [41] gewählten Grenzen entsprechen eher dem Vorschlag von Bühl [5], der ab r > 0,20 geringe, ab r > 0,50 mittelstarke und ab r > 0,70 starke Zusammenhänge als Interpretation vorschlägt. Unterschiede in der Höhe von Korrelationskoeffizienten wurden über Olkins z [42] mit dem Programm von Hemmerich [17] auf Signifikanz geprüft.

Zur Bestimmung von Unterschieden im Summenscore des AAQ-II‑P bei Patienten, die sich in demografischen und klinischen Merkmalen unterscheiden (Geschlecht, Schulabschluss, Chronifizierungsstadium, Schmerzgraduierung), wurden *t*-Tests für unabhängige Gruppen bzw. einfaktorielle Varianzanalysen mit Bonferroni-korrigierten Post-hoc-Tests gerechnet. Mögliche Alterseffekte wurden mittels Produkt-Moment-Korrelation überprüft, Zusammenhänge mit der Schulbildung wurden über Rangkorrelation nach Spearman abgebildet. Die Auswertungen erfolgten mit dem Programm SPSS (Version 22). Es wurden nur Patienten berücksichtigt, für die ein AAQ-II‑P-Gesamtwert vorlag.

## Ergebnisse

### Patientenstichprobe

Die meisten Patienten gaben als Hauptschmerz „Rückenschmerzen“ (39,3 %) an und waren dem Chronifizierungsstadium III (MPSS) zuzuordnen (60,6 %). In der Schmerzgraduierung nach von Korff hatten die Patienten mehrheitlich den höchsten Schmerzschweregrad 4 (51,5 %). Tab. 1 fasst soziodemografische und schmerzbezogene Merkmale des Patientenkollektivs zusammen, Tab. 2 zeigt deskriptive Statistiken der psychometrischen Verfahren.

**Table 1:** 

Merkmal	Ausprägung
*Geschlecht, n (%)*	
Männlich	38 (22,6)
Weiblich	130 (77,4)
*Alter in Jahren, M (SD)*	52,3 (10,2)
*Schulabschluss, n (%)*	
Kein Abschluss	4 (2,4)
Hauptschule	53 (32,3)
Mittlere Reife	72 (43,9)
Fachabitur	11 (6,7)
Abitur	24 (14,6)
*Hauptschmerz (MASK-S), n (%)*	
1 Kopfschmerz	10 (6,0)
2 Gesichtsschmerz	1 (0,6)
3 Schmerz bei Durchblutungsstörung	0 (0,0)
4 neurogener Schmerz	38 (22,6)
5 Rückenschmerz	66 (39,3)
6 Muskel‑, Gelenk‑, Knochenschmerz	45 (26,8)
7 viszeraler Schmerz	7 (4,2)
8 sonstiger Schmerz	1 (0,6)
*Schmerzchronifizierung (MPSS), n (%)*	
Stadium I	20 (14,6)
Stadium II	34 (24,8)
Stadium III	83 (60,6)
*Schmerz-Grading, n (%)*	
Grad 1	17 (10,2)
Grad 2	17 (10,2)
Grad 3	47 (28,1)
Grad 4	86 (51,5)

Abweichung *n* von 168 durch fehlende Werte

*MASK‑S* Multiaxiale Schmerzklassifikation – somatische Dimension, *MPSS* Mainz Pain Staging System

**Table 2:** 

	*N*	Range	M	SD
*AAQ-II‑P*				
Gesamtwert	168	8–49	29,89	9,38
*KIMS‑S*				
Beobachten	168	2–5	3,49	0,75
Beschreiben	168	1–5	3,33	0,90
Mit Aufmerksamkeit handeln	168	1–5	2,86	0,78
Akzeptieren ohne bewerten	168	1–5	3,50	0,93
*PCS*				
Hilflosigkeit	168	0–24	12,59	5,65
Rumination	168	0–16	8,45	3,88
Magnifikation	168	0–12	5,23	2,88
Gesamtwert	168	0–52	26,31	11,27
*BFI‑K*				
Extraversion	168	1–5	3,05	0,90
Verträglichkeit	168	2–5	3,26	0,77
Gewissenhaftigkeit	168	2–5	3,80	0,67
Neurotizismus	168	2–5	3,60	0,84
Offenheit	168	2–5	3,75	0,69
*HADS‑D*				
Angst	168	1–21	9,57	4,36
Depression	168	0–20	9,65	4,90
*SF-12*				
Körperliche Summenskala	168	16–58	30,98	8,97
Psychische Summenskala	168	16–64	39,27	11,54

*AAQ-II‑P* Acceptance and Action Questionnaire II – Pain, *KIMS‑S* Kentucky Inventory of Mindfulness Skills – Short Version, *PCS* Pain Catastrophizing Scale, *BFI‑K* Big Five Inventory – Kurzform, *HADS‑D* Hospital Anxiety and Depression Scale – Deutsch, *SF-12* Short-Form 12

### AAQ-II‑P: faktorielle Struktur und Itemanalyse

Von 163 Patienten wurden alle 7 Items des AAQ-II‑P beantwortet, ein Patient hatte 2 fehlende Werte und 4 Patienten hatten einen fehlenden Wert. Für diese Patienten wurden die fehlenden Werte durch den Mittelwert der beantworteten Items geschätzt.

**Figure 1:**
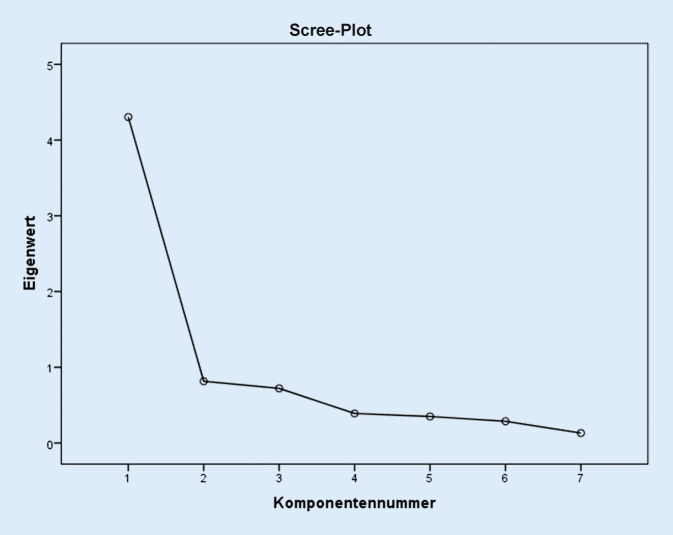

Die Faktorenanalyse legte nach Scree-Plot und Kaiserkriterium (Eigenwert > 1) eine Einfaktorenlösung nahe, durch die 61,5 % Varianz aufgeklärt wurde. Abb. 1 zeigt den Scree-Plot mit den Eigenwerten für jede Komponente.

Die interne Konsistenz der AAQ-II‑P-Summenskala (Cronbachs α) war 0,89. Die korrigierte Item-Skala-Korrelation (Trennschärfe) war für die Items mindestens *r* = 0,63. Tab. 3 fasst die Itemkennwerte des AAQ-II‑P zur Reliabilitätsanalyse und die Faktorenladungen der Items für die eindimensionale Lösung zusammen.

**Table 3:** 

	M	SD	Trennschärfe^a^	Faktorenladung
1) Meine Schmerzen machen es mir schwer, ein Leben zu führen, das ich wertschätzen kann	4,36	1,62	0,77	0,86
2) Ich habe Angst vor meinen Schmerzen	3,83	1,76	0,65	0,73
3) Ich befürchte, dass ich meine Schmerzen nicht kontrollieren kann	4,12	1,69	0,66	0,75
4) Meine Schmerzen hindern mich daran, ein erfülltes Leben zu haben	4,71	1,73	0,79	0,87
5) Schmerzen verursachen Probleme in meinem Leben	5,36	1,47	0,69	0,79
6) Die meisten Menschen scheinen ihr Leben besser im Griff zu haben als ich	3,61	1,85	0,67	0,75
7) Sorgen über meine Schmerzen stehen meinem Erfolg im Weg	4,01	1,87	0,63	0,73

Kodierung der Items von 1 bis 7

^a^Korrigierte Item-Skala-Korrelationen

### Konstruktvalidität des AAQ-II‑P

Die Häufigkeitsverteilung des AAQ-II‑P-Gesamtwerts wich nicht von einer Normalverteilung ab (*p* = 0,20). Männer hatten signifikant höhere Werte als Frauen (M = 33,15 ± 8,87 vs. M = 28,94 ± 9,35; *t*(166) = 2,47; *p* = 0,014), die Größe des Unterschieds lag im kleinen bis mittleren Bereich (*d* = 0,46).

Die varianzanalytischen Auswertungen zeigten, dass Patienten in unterschiedlichen MPSS-Chronifizierungsstadien sich signifikant in ihrem AAQ-II‑P-Gesamtwert unterschieden (*F*(2,134) = 5,3; *p* = 0,006; Eta_p_^2^ = 0,07). Dies traf auch für die Schmerzgraduierung nach von Korff zu (*F*(3,163) = 16,86, *p* < 0,001, Eta_p_^2^ = 0,24).

**Figure 2:**
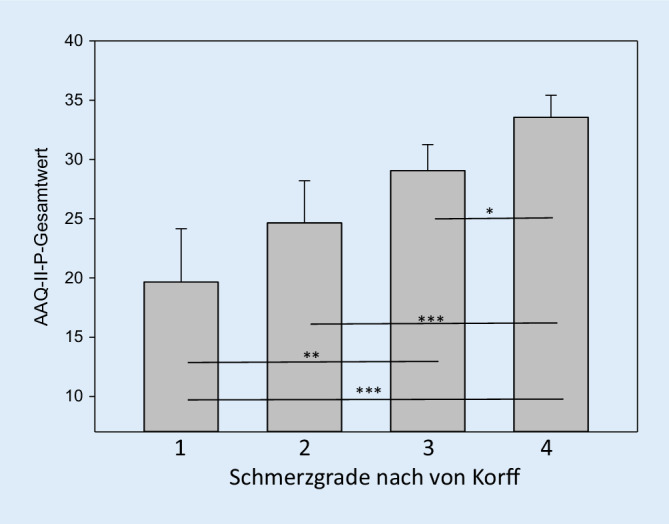

Abb. 2 zeigt die Ausprägung des AAQ-II‑P in Abhängigkeit von der Korff-Schmerzgraduierung.

Weder Schulbildung (*r*_*s*_ = −0,08) noch Alter korrelierte signifikant mit dem AAQ-II‑P-Gesamtwert (*r* = −0,002). Tab. 4 zeigt die Korrelationen zwischen dem AAQ-II‑P-Gesamtwert und den psychometrischen Verfahren.

**Table 4:** 

Verfahren	*N*	Korrelation
*Schmerz (NRS)*		
Schmerzintensität	167	0,47***
Schmerzbedingte Beeinträchtigung	166	0,58***
*KIMS‑S*		
Beobachten	168	−0,13
Beschreiben	168	−0,39***
Mit Aufmerksamkeit handeln	168	0,06
Akzeptieren ohne bewerten	168	−0,43***
*BFI‑K*		
Extraversion	168	−0,33***
Verträglichkeit	168	−0,07
Gewissenhaftigkeit	168	−0,36***
Neurotizismus	168	0,44***
Offenheit	168	−0,09
*PCS*		
Hilflosigkeit	168	0,76***
Rumination	167	0,59***
Magnifikation	168	0,61***
Gesamtwert	168	0,75***
*HADS‑D*		
Angst	168	0,66***
Depression	168	0,73***
*SF-12*		
Körperliche Summenskala	168	−0,32***
Psychische Summenskala	168	−0,58***

*AAQ-II‑P* Acceptance and Action Questionnaire II – Pain, *KIMS‑S* Kentucky Inventory of Mindfulness Skills – Short Form, *PCS* Pain Catastrophizing Scale, *BFI‑K* Big Five Inventory – Kurzform, *HADS‑D* Hospital Anxiety and Depression Scale – Deutsch, *SF-12* Short-Form 12

***p < 0,001

Die Korrelation der schmerzbezogenen Erlebensvermeidung (AAQ-II‑P Gesamtwert) und der schmerzbedingten Beeinträchtigung war signifikant höher als die Korrelation zur Schmerzintensität (*p* = 0,04). Signifikant negative Korrelationen fanden sich zu „Akzeptieren ohne bewerten“ und „Beschreiben“, hingegen bestand kein signifikanter Zusammenhang zu anderen Facetten der Achtsamkeit (KIMS-S-Subskalen „Beobachten“ und „Mit Aufmerksamkeit handeln“). Hohe positive Beziehungen lagen zu Schmerzkatastrophisieren vor (PCS-Summenwert und -Subskalen). In Bezug auf die Lebensqualität (SF-12) war die Beziehung zwischen schmerzbezogener Erlebensvermeidung und der Summenskala „psychische Gesundheit“ mit *p* = 0,004 signifikant höher als zu der Summenskala „körperliche Gesundheit“. Bei den psychologischen Persönlichkeitsmerkmalen (BFI-Subskalen) lag die höchste Beziehung zum Neurotizismus vor.

## Diskussion

Die Ergebnisse sprechen dafür, dass die deutsche Version des AAQ-II‑P ein eindimensionales psychometrisches Messinstrument ist, das schmerzbezogene Erlebensvermeidung reliabel erfasst.

### Dimensionalität und Reliabilität

Die interne Konsistenz der deutschen Version des AAQ-II‑P ist vergleichbar mit derjenigen der Originalversion [47]. Die einfaktorielle Lösung klärte mit 61,5 % sogar etwas mehr Varianz auf als bei der niederländischen Stichprobe (56,2 %). Die Hypothesen zur Dimensionalität und Reliabilität konnten bestätigt werden und zeigen zudem eine hohe Übereinstimmung mit Befunden zur nicht-schmerzbezogenen Ursprungsversion AAQ-II [3, 12], wobei dies auch für die Anwendung des AAQ-II bei Patienten mit chronischem Schmerz zutrifft [38, 51]. Der geringe Anteil fehlender Werte legt nahe, dass das Verfahren keinen Inhalt hat, zu dem Patienten keine Auskunft geben können oder möchten. Die fehlende Abweichung von einer Normalverteilung spricht ebenso für positive psychometrische Eigenschaften des Verfahrens. Hinsichtlich der Mittelwerte ist die Auswertung mit den Daten von Reneman et al. [47] vergleichbar.^1^ Das Lübecker Patientenkollektiv liegt mit einem Skalengesamtwert von M = 29,9 geringfügig über dem Wert der niederländischen Stichprobe (M = 28,0). Männliche Schmerzpatienten berichteten höhere Werte in schmerzbezogener Erlebensvermeidung, was sich ebenfalls in der niederländischen Version findet. Geschlechtsunterschiede in dieser Richtung zeigen sich interessanterweise nicht, wenn nach schmerzunspezifischer Erlebensvermeidung (erhoben mittels AAQ-II) gefragt wird. Hier finden sich sowohl Hinweise auf fehlende Unterschiede [3] wie auch solche auf höhere Werte bei Frauen [25]. Eine mögliche Erklärung liegt in der thematischen Fokussierung auf Schmerz, die ausschließlich mit dem AAQ-II‑P vorgenommen wird. Denkbar wäre, dass Männer eine höhere Neigung haben, Schmerz nicht zu akzeptieren [49].

### Zusammenhang mit Schmerzkatastrophisieren und Achtsamkeit

Es wurde vermutet, dass der Summenscore des AAQ-II‑P schwach oder mittelstark mit Skalen anderer psychometrischer Verfahren korreliert, die mit dem Konzept der schmerzbezogenen Erlebensvermeidung vergesellschaftet sind. Diese Hypothese war an der Formulierung von Reneman et al. angelehnt [47], die eine Korrelation von *r* = 0,59 zwischen Schmerzkatastrophisieren (PCS) und dem AAQ-II‑P berichteten. Mit *r* = 0,75 fanden wir ebenso eine deutlich überzufällige Beziehung zwischen PCS und AAQ-II‑P. Dies war erwartbar, da sich die Verfahren inhaltlich zum Teil überschneiden (z. B. AAQ-II‑P : „Ich befürchte, dass ich meine Schmerzen nicht kontrollieren kann“; „Ich habe Angst vor meinen Schmerzen“. PCS: „Es gibt nichts, was ich tun kann, um meine Schmerzen zu lindern“; „Ich bekomme Angst, dass die Schmerzen noch stärker werden“). Schmerzbezogene Erlebensvermeidung steht dagegen in keiner oder nur geringer Beziehung zu Aspekten der Achtsamkeit, wie sie mit dem KIMS‑S erfasst werden. Es zeigten sich plausible, in der Höhe aber nur geringe Zusammenhänge mit der Fähigkeit, den eigenen Zustand in Worte zu fassen (Skala „Beschreiben“) und der Fähigkeit, Gefühle und Gedanken zu akzeptieren (Skala „Akzeptieren ohne bewerten“). Mit einem schmerzbezogenen Messverfahren für Akzeptanz (z. B. dem CPAQ) könnte ein deutlich stärkerer Zusammenhang zu schmerzbezogener Erlebensvermeidung bestehen. Keine signifikanten Beziehungen fanden sich zu der Fähigkeit, beim eigenen Handeln konzentriert zu sein (Skala „Handeln mit Aufmerksamkeit“), und zu der Fähigkeit, körperliche Empfindungen zu beobachten (Skala „Beobachten“). Das strikte Vermeiden schmerzbezogener Reize steht demnach in keinem Zusammenhang zu einem generell bewussten, gegenwärtigen Erleben und Beobachten der (nicht ausschließlich schmerzassoziierten) Umwelt. Die fehlende überzufällige Beziehung zur Skala „Beobachten“ wurde schon für den AAQ-II berichtet [8] und ist auch für den AAQ-II‑P naheliegend, da die meisten zugehörigen Items die Empfindung von äußeren Reizen beschreiben (z. B. Beobachten und Empfindungen von Sonnenschein auf dem Gesicht, von Wasser beim Duschen, von Vogelgezwitscher).

Zur Abgrenzung und zum genaueren Verständnis der Konstruktvalidität des AAQ-II‑P sollten zukünftige Untersuchungen weitere konzeptnahe schmerzunspezifische Verfahren einschließen (z. B. das White Bear Suppression Inventory [WBSI] [56]) sowie konzeptnahe schmerzspezifische Verfahren (z. B. Tampa Scale for Kinesiophobia [TSK] [55], Psychological Inflexibility in Pain Scale [PIPS] [2], Chronic Pain Acceptance Questionnaire [CPAQ] [41]). Dadurch ließe sich auch der entgegengesetzte Pol von schmerzbezogener Erlebensvermeidung klarer definieren.

### Zusammenhang mit Schmerzintensität, Schmerzbeeinträchtigung, Angst, Depressivität und Lebensqualität

Wenn schmerzbezogene Erlebensvermeidung für die Entwicklung oder Aufrechterhaltung chronischer Schmerzen bedeutsam ist, müsste diese mit Merkmalen assoziiert sein, die typischerweise ausgeprägter bei chronischen Schmerzpatienten sind [9, 22, 23]. Entsprechend wurde in der dritten Hypothese angenommen, dass der AAQ-II‑P positive Beziehungen zu Schmerzintensität, schmerzbedingten Beeinträchtigungen, Angst, Depressivität und (schlechter) gesundheitsbezogener Lebensqualität aufweist. Die Ergebnisse bestätigten diese Annahmen: Signifikante Korrelationen zu Intensität und Beeinträchtigung durch Schmerzen (NRS-Angaben), zu Angst und Depressivität (HADS-D) sowie zu den Summenskalen der gesundheitsbezogenen Lebensqualität (SF-12) entsprachen der formulierten Richtung und wiesen zwischen 10 und 53 % gemeinsame Varianz mit dem AAQ-II-P auf. Besonders hoch waren die Beziehungen zu Aspekten des psychischen Befindens (HADS‑D und „psychische Gesundheit“ von SF-12). Ähnlich deutliche Assoziationen zu Depressions- und Angstsymptomen sowie allgemein zu psychischer Belastung wurden bereits für allgemeine Erlebensvermeidung/psychische Inflexibilität (gemessen mit dem AAQ-II) berichtet (0,49 ≤ *r* ≤ 0,73; [3, 12]). Des Weiteren deuten die Ergebnisse darauf hin, dass der Zusammenhang zwischen schmerzbezogener Erlebensvermeidung und schmerzbedingter Beeinträchtigung ausgeprägter ist als der Zusammenhang zur Schmerzintensität. So fällt die Korrelation des AAQ-II‑P mit der schmerzbedingten Beeinträchtigung signifikant höher aus als die zur Schmerzintensität.

### Zusammenhang mit Schmerzgrad nach von Korff und Schmerzchronifizierung

Analog finden sich bei der Schmerzgraduierung nach von Korff in Abhängigkeit von der vorliegenden Beeinträchtigung abgestufte AAQ-II‑P-Werte (Grad 3 < Grad 4; *p* < 0,001), während sich Patienten mit geringen vs. starken Schmerzen bei geringer schmerzbedingter Beeinträchtigung nicht unterscheiden (Grad 1 = Grad 2, *p* = 0,475). Es ist möglich, dass schmerzbedingte Vermeidung eher mit schmerzbedingter Beeinträchtigung einhergeht als mit erhöhter Schmerzintensität. Dies unterstreicht die Relevanz von schmerzbezogener Erlebensvermeidung als Zielvariable in der schmerzpsychotherapeutischen Behandlung. Obwohl unsere Befragung ausschließlich assoziative Daten liefert, erscheint die Annahme plausibel, dass sich als Folge von schmerzbezogener Erlebensvermeidung bei Menschen mit chronischem Schmerz schmerzbedingte Beeinträchtigungen und negatives psychisches Befinden entwickeln. Aktuelle Analysen mit der deutschen Form des AAQ-II im Rahmen einer randomisierten, kontrollierten Studie (RCT) zur Wirksamkeit eines internetbasierten ACT-Programms bei Patienten mit chronischem Schmerz zeigen auf, dass psychologische Flexibilität eine Mediator- und Moderatorfunktion für Interventionseffekte auf Schmerzparameter wie Schmerzinterferenz und gesundheitsbezogene Lebensqualität einnimmt [32, 45]. Dies könnte ein wichtiger Gegenstand zukünftiger Untersuchungen sein.

Der Zusammenhang zwischen MPSS-Stadium und AAQ-II‑P in dieser Stichprobe war zwar positiv, doch gering (*r*_*S*_ = 0,25). Dies lässt sich auf die vergleichsweise geringe Varianz des Chronifizierungsgrads zurückführen. Eine multizentrische Auswertung mit dem Dokumentationssystem KEDOQ legt für Schmerzambulanzen in Deutschland im Mittel einen Anteil von 47,5 % an Patienten mit höchstem Chronifizierungsstadium nahe [23]. In der hier untersuchten Stichprobe lag der Anteil mit 60,6 % deutlich höher.

### Zusammenhang mit Persönlichkeitsmerkmalen

Übereinstimmend mit der vierten Hypothese, dass schmerzbezogene Erlebensvermeidung nur wenig mit Persönlichkeitsmerkmalen verbunden ist, fanden sich nur bedeutungslose oder geringe bis maximal mittlere Beziehungen zu den „Big 5“. Am höchsten und in plausibler Richtung lag die Beziehung für emotionale Labilität/Neurotizismus vor (*r* = 0,44). Hier fehlen Vergleichsdaten mit Schmerzpatienten aus anderen Studien. Für den AAQ-II wurden in der Vergangenheit höhere Korrelationen mit Neurotizismus berichtet, wie *r* = 0,57 [12]. Der Unterschied zu unseren Befunden lässt sich möglicherweise zurückführen auf die schmerzbezogenen Inhalte des AAQ-II‑P (die des AAQ-II sind schmerzunspezifisch) und die schmerzunspezifischen Inhalte der Items in Persönlichkeitsfragebögen.

### Störungsspezifische Erlebensvermeidung

Der AAQ-II‑P fügt sich ein in eine Reihe von störungsspezifischen Versionen des AAQ. Solche wurden bislang unter anderem für Substanzmissbrauch [33], soziale Phobie [34], Traumata [44] und kardiovaskuläre Erkrankungen [52] entwickelt. Ein aktuelles Review [43] fasst die bereichsspezifischen Varianten zusammen und liefert Hinweise auf deren teststatistische Überlegenheit gegenüber der generischen Erhebung von Erlebensvermeidung und psychologischer Inflexibilität in spezifischen Populationen. In einer weiteren Metaanalyse moderiert die Art des Messinstruments (spezifisch vs. unspezifisch) den Einfluss von Furchtvermeidung auf Schmerzintensität, mit größeren Effektstärken bei der Verwendung schmerzspezifischer Erhebungsverfahren [30].

## Limitationen der Studie

Die Abbildung der Konstruktvalidität ist eingeschränkt auf die Vergleiche mit den in der Umfrage eingeschlossenen Instrumenten. Eine Abfrage weiterer einschlägiger Instrumente zur Messung verwandter Konstrukte, wie beispielsweise Vermeidung (WBSI, TSK), psychischer Flexibilität (PIPS) oder Schmerzakzeptanz (CPAQ), wäre wünschenswert. Weitere Einschränkungen ergeben sich dadurch, dass die hier vorgelegten Befunde ausschließlich auf monozentrisch erfassten Querschnittsdaten basieren. Benötigt werden nun Verlaufsdaten, um bewerten zu können, inwieweit sich der AAQ-II‑P z. B. als Outcome-Parameter oder zur Prädiktion von Behandlungserfolgen bei Schmerzpatienten eignet. Wir vermuten hier ein hohes Potenzial.

## Fazit für die Praxis

- Der AAQ-II‑P ist ein mit sieben Items äußerst ökonomischer, einfach auszufüllender und reliabler Fragebogen, der lizenzfrei für wissenschaftliche Fragestellungen und Patientenversorgung verwendet werden kann. Das Verfahren quantifiziert „schmerzbezogene Erlebensvermeidung“ bzw. „Schmerzakzeptanz“.
- Das Verfahren ist nicht an ACT gebunden und kann in anderen therapeutischen Ansätzen wie der kognitiven Verhaltenstherapie oder im Kontext einer interdisziplinären multimodalen Schmerztherapie verwendet werden. Hier kann die Reduktion schmerzbezogener Erlebensvermeidung eine Zielformulierung der Behandlung sein.

## Footnotes

1 Allerdings muss dabei folgende Anmerkung beachtet werden: Die Autoren geben im Text eine Itemskalierung von 0 bis 6 an und beziffern die Spanne ihrer Skalensumme mit 0–42. Das widerspricht jedoch der Darstellung des Verfahrens und dem Auswertungshinweis im Anhang der Publikation (Skalierung 1–7) sowie der im Text dargestellten Häufigkeitsverteilung (Spanne 7–49). Wir haben die Auswertung des AAQ-II‑P-Gesamtwerts nach Angaben der Testbeschreibung im Anhang der Arbeit von Reneman et al. [47] und in Übereinstimmung mit der Beschreibung bei Bond et al. [3] vorgenommen (1–7).
